# Interferon-stimulated gene 20 kDa protein serum levels and clinical outcome of hepatitis B virus-related liver diseases

**DOI:** 10.18632/oncotarget.25559

**Published:** 2018-06-12

**Authors:** Hoang Van Tong, Nghiem Xuan Hoan, Mai Thanh Binh, Dao Thanh Quyen, Christian G. Meyer, Le Huu Song, Nguyen Linh Toan, Thirumalaisamy P. Velavan

**Affiliations:** ^1^ Institute of Biomedicine and Pharmacy, Vietnam Military Medical University, Hanoi, Vietnam; ^2^ Department of Pathophysiology, Vietnam Military Medical University, Hanoi, Vietnam; ^3^ Institute of Tropical Medicine, University of Tübingen, Tübingen, Germany; ^4^ 108 Military Central Hospital, Hanoi, Vietnam; ^5^ Vietnamese-German Center of Excellence in Medical Research, Hanoi, Vietnam; ^6^ Duy Tan University, Da Nang, Vietnam

**Keywords:** interferon-stimulated gene, ISG20 levels, ISG20 expression, viral hepatitis, hepatocellular carcinoma

## Abstract

Interferon-stimulated gene 20 kDa protein (ISG20) with 3’ to 5’ exonuclease activity mainly targeting single-stranded RNA plays an important role in immune responses against various infectious pathogens, including hepatitis viruses. ISG20 levels were measured by ELISA assays in sera of 339 hepatitis B-virus (HBV) infected patients and 71 healthy individuals and were correlated with clinical and laboratory parameters. *ISG20* mRNA was quantified by qRT-PCR in 30 pairs of hepatocellular carcinoma (HCC) tumour and adjacent non-tumour liver tissues. ISG20 levels were significantly elevated in HBV patients compared to healthy controls (*P*<0.0001). In the patient group, varying ISG20 levels were associated with different forms of HBV-related liver diseases. ISG20 levels were higher in patients with HCC compared to those without HCC (*P*<0.0001), and increased according to the stages of HCC (*P*<0.0001). ISG20 mRNA expression was up-regulated in tumour tissues compared to the expression in adjacent non-tumour tissues (*P*=0.017). Importantly, ISG20 levels were strongly correlated with the levels of AST, ALT, total and direct bilirubin among HCC patients (Pearson’s r = 0.43, 0.35, 0.34, 0.3; *P*<0.0001, respectively). Although differences between liver cirrhosis (LC) and non-LC patients were not observed, ISG20 levels were elevated according to the progression of cirrhosis in patients with LC plus HCC (*P*=0.005). In conclusions, ISG20 levels are induced by HBV infection and significantly associated with progression and clinical outcome of HBV-related liver diseases, especially in patients with HCC. ISG20 might be a potential indicator for liver injury and the clinical outcome in HBV-related HCC.

## INTRODUCTION

Hepatitis B virus (HBV) infection affects approximately 257 million people worldwide and causes 887.000 deaths annually due to its complications. Regions with high prevalences of HBV infection include sub-Saharan Africa, Asia and some parts of the Americas, with infection rates in some regions being more than 8% of the population [1]. HBV infection is a leading cause of liver diseases, including acute self-limiting and fulminant hepatitis, chronic hepatitis B (CHB), liver cirrhosis (LC) and hepatocellular carcinoma (HCC). Rates of progression to LC in chronic HBV carriers are estimated to be 10% per year [2], and the risk of HCC development in chronic HBV carriers is 100-fold higher compared to HBV-negative individuals [3]. In patients with HBV-related LC, the 5-year cumulative occurrence of HCC is about 15% in highly endemic areas such as sub-Saharan Africa and Asia [2]. Annually, more than 500,000 new HCC cases are diagnosed worldwide [3]. HCC can develop on the basis of chronic liver injury, inflammation, and cirrhosis through a complex of mechanisms, and the risk of HCC development in chronic HBV carriers is directly related to high levels of HBV replication in hepatocytes [3, 4].

Liver injury during HBV infection is mainly due to the activity of HBV-specific T cells, and the progression of HBV-related liver diseases is a consequence of the interaction between viral factors and host immune responses [5]. Host immune factors play a central role in the pathogenesis of HBV infection via activation of the cytokine system that triggers the first line of defence against infections [6]. In response to HBV infection, interferons are produced by the host, bind to cell surface receptors and activate cellular signalling pathways, in particular the JAK/STAT signalling pathway (Janus kinase / signal transducer and activator of transcription) in order to inhibit virus replication. Thus, induction of interferons is essential for the host defence against viral infections, including viral hepatitis [7].

Interferon-stimulated genes (ISGs) encode a large group of proteins induced mainly by interferons and regulating host immune responses. ISGs are actively involved in controlling infections through multiple mechanisms, including directly affecting certain stages of the development and replication of pathogens [8]. Among the ISG family, the interferon-stimulated gene 20 kDa protein (ISG20), also known as human estrogen regulated transcript 45 (HEM45), is a protein belonging to the DEDDh subgroup of the DEDD exonuclease superfamily, defined by three aspartates (D) and one glutamate (E) [9, 10]. ISG20 is encoded by the *ISG20* gene located on chromosome 15q26.1 (NC_000015.10), and is induced by both type I and II interferons [9], double-stranded RNA [11], estrogen and thyroid hormones [12, 13], as well as by virus-encoded transcription factors [14]. ISG20 displays a strong 3’ to 5’ exonuclease activity, mainly targeting single-stranded rather than double-stranded RNA or single-stranded DNA [11]. ISG20 plays a central role in host immune responses against various viruses [8, 15]. In particular, ISG20 has been shown to inhibit the replication of multiple positive-stranded RNA viruses, including yellow fever virus (YFV), bovine viral diarrhoea virus (BVDV), vesicular stomatitis virus (VSV), encephalomyocarditis virus (EMCV), influenza virus and human immunodeficiency virus type 1 (HIV-1) [16–18].

With regard to hepatitis viruses, ISG20 does not only exhibit a strong antiviral activity against RNA viruses such as hepatitis A and C viruses (HAV, HCV), but also inhibits HBV replication by directly targeting the epsilon stem-loop structure of HBV-RNA [19, 20]. A recent study has shown that expression levels of the ISG20 protein and *ISG20* mRNA were higher in livers of chronic hepatitis B patients responding well to interferon-alpha treatment compared to chronic hepatitis B non-responders [21]. The antiviral activities of ISG20 against hepatitis viruses and its differential expression pattern in response to interferon-alpha treatment suggest a crucial role of ISG20 as an immune effector and regulatory molecule in the outcome of HBV infection. Although ISG20 effectively impairs replication of many viruses, particularly that of HAV, HBV and HCV, the relevance of ISG20 serum levels in patients with viral hepatitis and the role of ISG20 in the pathogenesis and clinical outcome of HBV infection has not been established so far. This study investigates ISG20 serum levels in Vietnamese patients with HBV-related liver diseases and their association with clinical progression of HBV infection.

## RESULTS

### Clinical characteristics of patients with HBV-related liver diseases

The main demographic, clinical and laboratory characteristics of the 339 patients with HBV-related liver diseases are summarized in Table 1. The staging of LC and HCC based on Child-Pugh scores and the BCLC classification are also presented. Red blood cell, white blood cell and platelet counts were lower in LC patients compared to the other groups (*P*<0.001). AST levels were higher among patients with LC compared to those without LC, while ALT levels were higher in CHB patients compared to the other groups (*P*<0.0001). Similarly, total and direct bilirubin levels were higher in patients with LC compared to the non-LC group (*P*<0.0001). Albumin and prothrombin levels were lower in LC patients than in the other groups (*P*<0.0001). AFP levels were significantly increased in patients with HCC compared to those without HCC (*P*<0.0001). In addition, there were significant differences in the number of patients presenting with distinct Child-Pugh stages among LC patients, those with only HCC and those with LC plus HCC. In contrast, no difference in staging frequencies according to BCLC classification was observed between patients with only HCC and those with LC plus HCC (Table 1).

**Table 1:** Characteristics of patients with HBV-related liver disease segregated according to clinical status

Characteristics	CHB (n=100)	LC (n=79)	HCC (n=79)	LC+HCC (n=81)	HC (n=71)	*P* value
Age (median, range)	42 (17-85)	58 (29-82)	54 (16-84)	62 (29-80)	28 (23-46)	< 0.0001
Gender (male/female)	79/21	64/15	78/1	80/1	31/40	< 0.0001
HBeAg (pos/neg)	21/65 ^#^	0/23 ^#^	0/7 ^#^	0/4 ^#^	NA	NA
Anti-HBe (pos/neg)	55/29 ^#^	20/3 ^#^	4/3 ^#^	4/0 ^#^	NA	NA
Anti-HBc (pos/neg)	82/1 ^#^	23/0 ^#^	7/0 ^#^	4/0 ^#^	NA	NA
RBC (x10^3^/ml)	4.8 (3-6)	4.02 (2.5-5.7)	4.6 (3.4-6.8)	4.3 (2.4-6)	NA	< 0.0001
WBC (x10^6^/ml)	6.7 (4.5-13.9)	5.6 (2.4-18)	6 (3-11.6)	5.8 (2.7-17)	NA	0.001
PLT (x10^3^/ml)	201 (61-388)	91 (22-325)	195 (102-382)	114 (35-261)	NA	< 0.0001
AST (IU/L)	68.5 (15-1732)	77.5 (18-1221)	55 (17-950)	74.5 (25-670)	NA	0.008
ALT (IU/L)	75.5 (9-1876)	55 (8-692)	40 (12-262)	50 (11-805)	NA	< 0.0001
Total-Bilirubin (mg/dl)	16 (7-452)	25.5 (8.8-501)	16 (8-134)	23 (9-185)	NA	< 0.0001
Direct-Bilirubin (mg/dl)	6 (1-298)	10 (0.4-244)	5 (1-80)	11 (0.4-59)	NA	< 0.0001
Albumin (g/L)	42 (9.8-67)	31 (3.3-47)	40 (29-49)	36 (21-46)	NA	< 0.0001
Prothrombin (%)	92.6 (41-257)	59 (13-120)	82 (54-135)	74 (19.6-124)	NA	< 0.0001
HBV-DNA (log10 copies/ml)	6.1 (2-10.3)	5.2 (2-8.7)	4.3 (2-8.7)	4.8 (2-9.5)	NA	< 0.0001
Alpha fetoprotein (IU/L)	5 (5-178)	7.05 (1.2-300)	65 (1.07-300)	134 (1.55-350)	NA	< 0.0001
Liver cirrhosis stage:						< 0.0001
*Child-Pugh A (n,%)*	NA	27 (34%) ^§^	74 (94%) ^§^	42 (52%)	NA	
*Child-Pugh B (n,%)*	NA	26 (33%) ^§^	5 (6%) ^§^	33 (41%)	NA	
*Child-Pugh C (n,%)*	NA	19 (24%) ^§^	0 (0%) ^§^	6 (7%)	NA	
HCC stage						NS
*Stage A (n,%)*	NA	NA	37 (47%) ^§^	28 (35%) ^§^	NA	
*Stage B (n,%)*	NA	NA	39 (49%) ^§^	39 (48%) ^§^	NA	
*Stage C (n,%)*	NA	NA	1 (1%) ^§^	3 (4%) ^§^	NA	
*Stage D (n,%)*	NA	NA	0 (0%) ^§^	6 (7%) ^§^	NA	
**IGS20 levels (ng/mL)**	16.4 (1.82-56.7)	13.9 (0.87-178.6)	21.2 (3.01-81.3)	24.5 (3.4-250)	9.6 (0.82-25.7)	< 0.0001

Abbreviations: CHB, chronic hepatitis B; LC, liver cirrhosis; HCC, hepatocellular carcinoma; LC+HCC, patients with LC plus HCC; HC, healthy control; RBC, red blood cells; WBC, white blood cells; PLT, platelets; AST and ALT, aspartate and alanine amino transferase; AFP, alpha-fetoprotein; IU, international unit; NA, not applicable and/or not available; NS, not significant. Values given are medians and ranges. *P* values were calculated by Chi-squared test and Kruskal-Wallis test where appropriate to compare variables among different groups. (^#^): Only data available. (^§^): Only patients with clear classification, Child-Pugh score and categories for Barcelona clinic liver cancer (BCLC) staging available.

### ISG20 levels in healthy controls and in patients with HBV-related liver diseases

**Figure 1:**
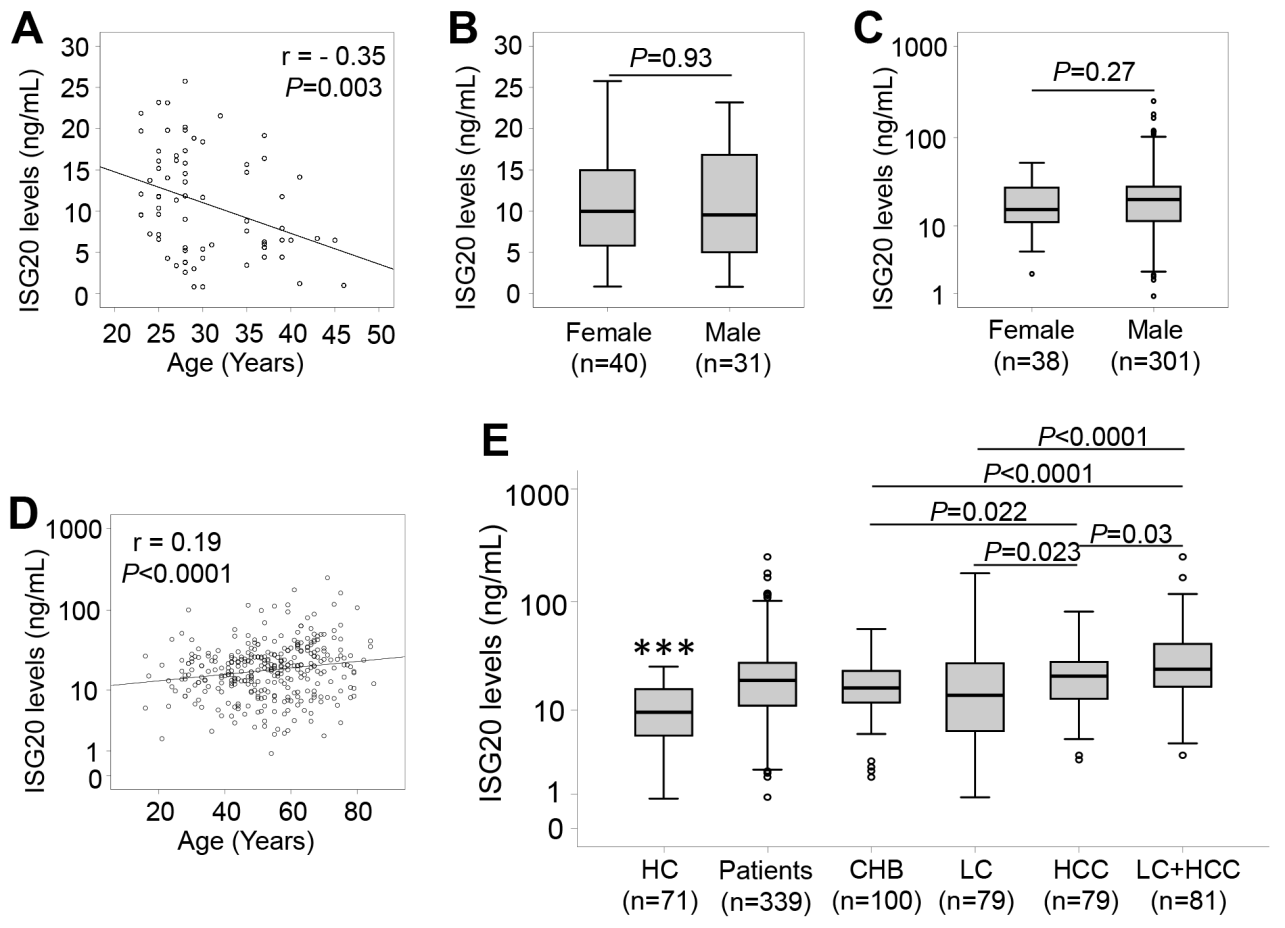
ISG20 levels in patients with HBV-related liver diseases and in healthy controls ISG20 levels were measured in all study subjects, correlated with age by using the Pearson correlation coefficient test, and compared between groups by using the Mann-Whitney-Wilcoxon test. **(A)**: Correlation of ISG20 levels with age in healthy controls. **(B)**: Comparison of ISG20 levels between male and female in healthy controls. **(C)**: Comparison of ISG20 levels between male and female in HBV patients. **(D)**: Correlation of ISG20 levels with age in HBV patients. **(E)**: ISG20 levels in different subgroups of patients with HBV-related liver diseases. HC, healthy controls; CHB, chronic hepatitis B; LC, patients with liver cirrhosis; HCC, patients with hepatocellular carcinoma; LC+HCC, patients with both liver cirrhosis and hepatocellular carcinoma. (^***^): *P*<0.0001 for comparison with other groups.

ISG20 levels were measured in patients with HBV-related liver diseases and in controls. In healthy individuals, we observed a median of 9.6 ng/ml, and ISG20 levels were negatively correlated with age (r = - 0.35, *P*=0.003), while there was no difference of ISG20 levels between males and females (*P*=0.93) (Figure 1A and 1B). In patients, we observed a median of 16.4 ng/mL in CHB patients, 13.9 ng/ml in LC patients, 21.2 ng/ml in patients with only HCC and 24.5 ng/ml in patients with LC plus HCC. We found a weak positive correlation of ISG20 levels with age (r = 0.19, *P*<0.0001), whereas ISG20 levels did not differ between male and female patients (*P*=0.27) (Figure 1C and 1D). These results indicate that age and the presence of HBV-related liver diseases influence the serum levels of ISG20.

The results of the comparisons show that ISG20 levels were significantly elevated in patients with HBV-related liver diseases compared to the controls (19.3 ng/ml vs. 10.9 ng/ml, *P*<0.0001) (Figure 1E), indicating that ISG20 levels are modulated due to HBV infection. In the patient group, ISG20 levels were lower in CHB patients compared to patients with only HCC (16.4 ng/mL vs. 21.2 ng/ml, *P*=0.022) and those with LC plus HCC (16.4 ng/mL vs. 24.5 ng/ml, *P*<0.0001). Increased ISG20 levels were observed in patients with only HCC (21.2 ng/ml, *P*=0.023) and those with LC plus HCC (24.5 ng/ml, *P*=0.002) when compared to LC patients (13.9 ng/ml). ISG20 levels were also higher among patients with LC plus HCC compared to those with only HCC (24.5 ng/ml vs. 21.2 ng/ml, *P*=0.03). However, no significant difference of ISG20 levels between CHB and LC patients was observed (16.4 ng/mL vs. 13.9 ng/ml, *P*>0.05) (Figure 1E).

### ISG20 levels and HBV-related LC

**Figure 2:**
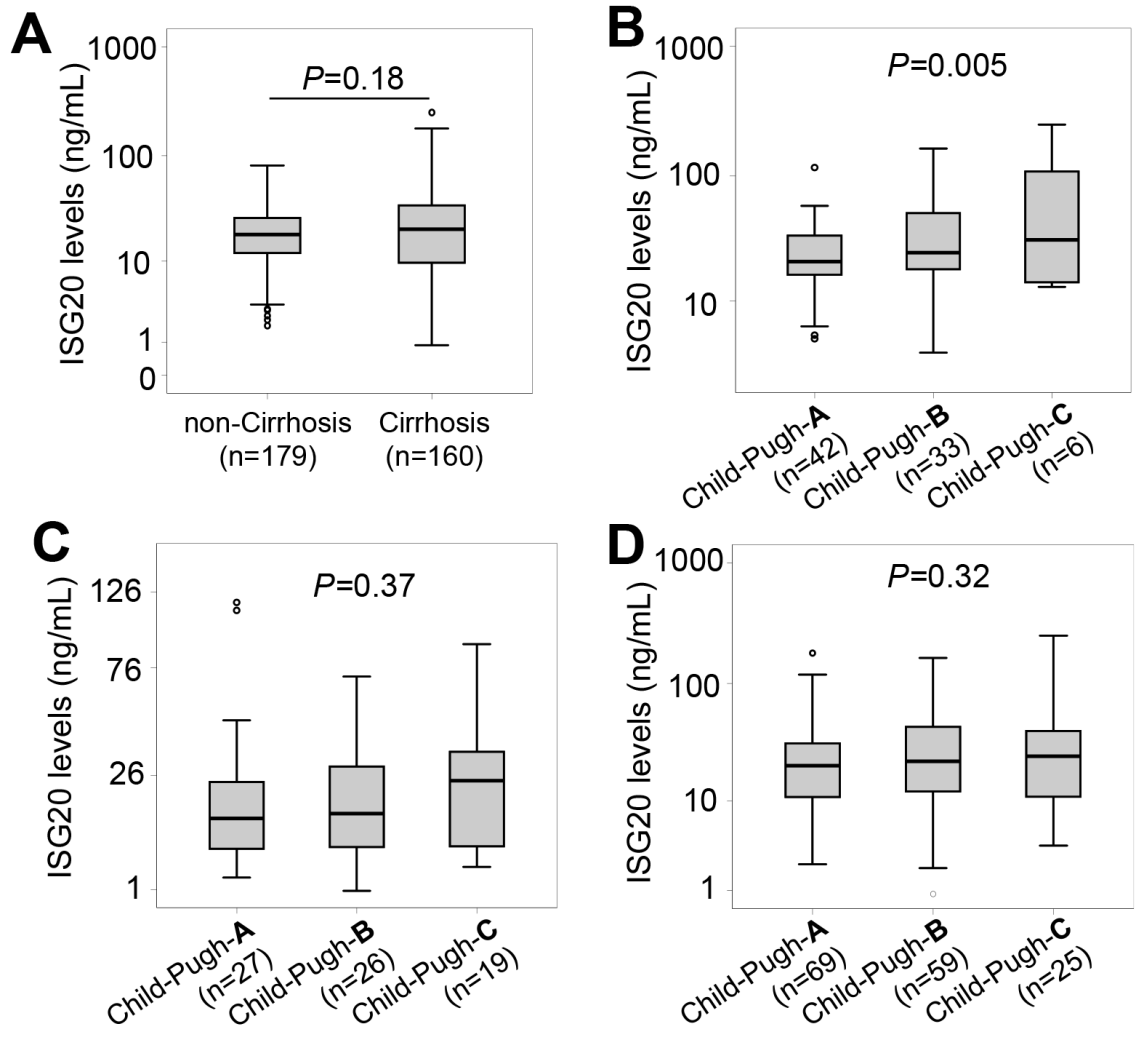
ISG20 levels in patients with HBV-related LC **(A)**: Comparison of ISG20 levels between patients with and without liver cirrhosis (LC). Patients with HCC were classified based on Child-Push scores, and ISG20 levels were compared between these classified subgroups. **(B)**: In patients with both LC and HCC, **(C)**: In LC patients with only LC; **(D)**: In all patients with only LC and those with LC plus HCC. *P* values were calculated by using Mann-Whitney-Wilcoxon test or linear regression adjusted for age and gender where appropriate.

We stratified the patients into subgroups with and without LC. ISG20 levels were not different between patients with and without LC (18.3 ng/ml vs. 20.6 ng/ml, *P*=0.18) (Figure 2A). We then classified LC patients and patients with LC plus HCC into the subgroups of Child-Pugh-A, Child-Pugh-B and Child-Pugh-C. ISG20 levels were increased according to the stage of LC in patients with LC plus HCC (*P*=0.005) (Figure 2B), but not in LC-only patients as well as in patients with LC (LC only patients and those with LC plus HCC) (Figure 2C and 2D).

### ISG20 levels and HBV-related HCC

**Figure 3:**
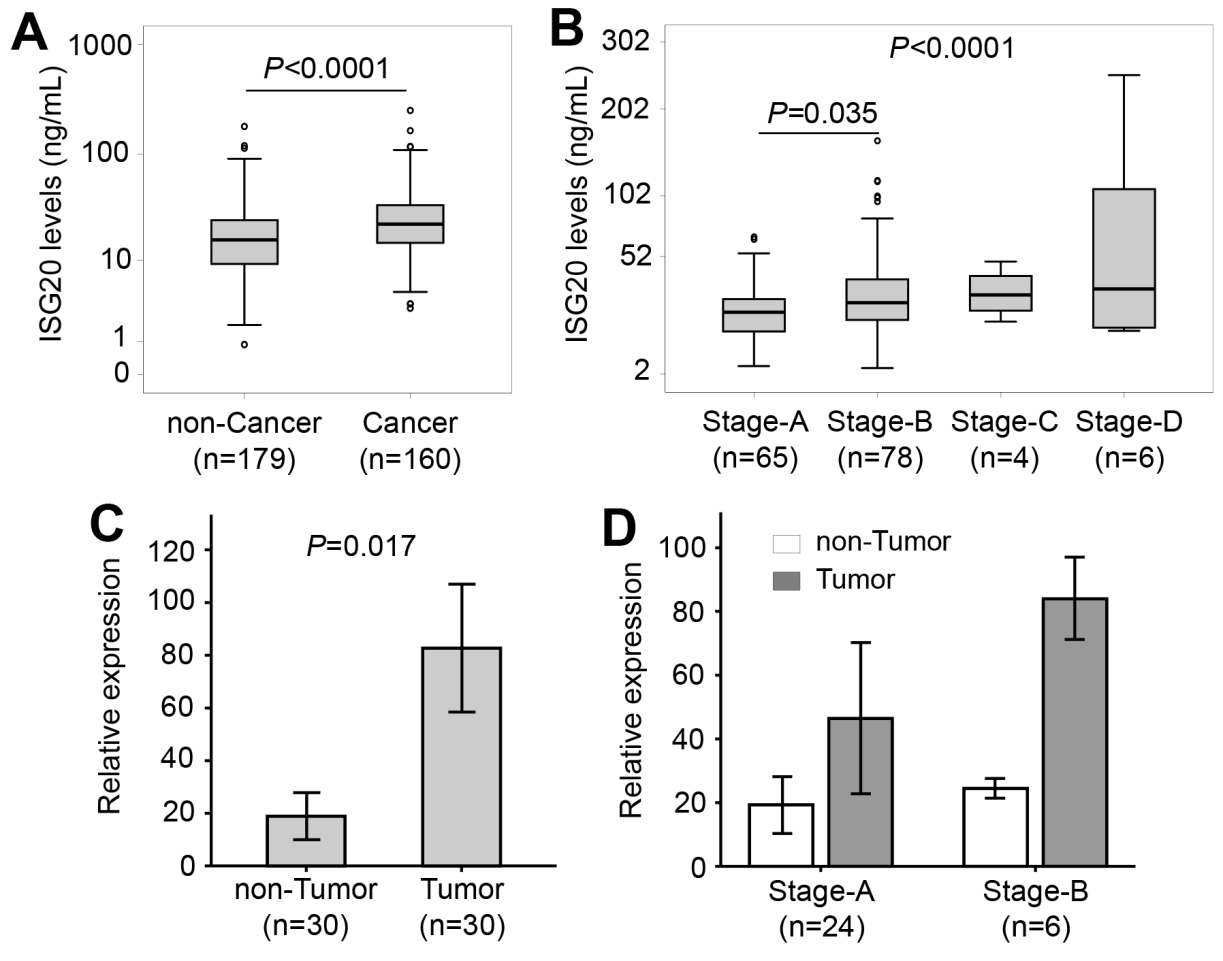
ISG20 serum and *ISG20* mRNA levels in patients with HBV-related LC **(A)**: Comparison of ISG20 levels between patients with and without hepatocellular carcinoma (HCC). **(B)**: Patients with HCC were classified based on categories for Barcelona clinic liver cancer (BCLC) staging, and ISG20 levels were compared between these classified subgroups. *P* values were calculated by using Mann-Whitney-Wilcoxon test or linear regression adjusted for age and gender where appropriate. **(C)**: Comparison of *ISG20* mRNA expression in tumour and adjacent non-tumour tissues. *P* values were calculated using Paired-samples *t*-test. **(D)**: *ISG20* mRNA expression in stage-A and stage-B tumour tissues and in adjacent non-tumour tissues.

Patients were categorized into subgroups with and without HCC. Patients with HCC had increased ISG20 levels compared to patients without HCC (22.4 vs. 15.8 ng/ml, *P*<0.0001) (Figure 3A). When further classifying the patients with HCC into subgroups based on the BCLC staging system, ISG20 levels were increased according to BCLC stages (*P*<0.0001). In particular, ISG20 levels were lower among stage A than stage B patients (*P*=0.035) (Figure 3B). These results indicate that ISG20 levels are associated with the occurrence and, most likely, progression of HBV-related HCC. Furthermore, we examined expression of *ISG20* mRNA in tumour and adjacent non-tumour tissues. The relative expression of *ISG20* mRNA was significantly up-regulated in tumour tissues compared to adjacent non-tumour tissues (*P*=0.017) (Figure 3C). We then examined whether the expression of *ISG20* mRNA was associated with the development of HCC by correlating *ISG20* mRNA expression with BCLC stages. *ISG20* mRNA expression was higher in stage-B tumour tissues compared to that in stage-A HCC tissues. A similar trend was seen when *ISG20* mRNA expression was compared between the non-tumour tissues obtained from stage-A and stage-B HCC patients; however, the difference did not reach statistical significance (Figure 3D). These results indicate that ISG20 expression is associated with HBV-related HCC.

### ISG20 levels and clinical outcome of HBV-related liver diseases

**Figure 4:**
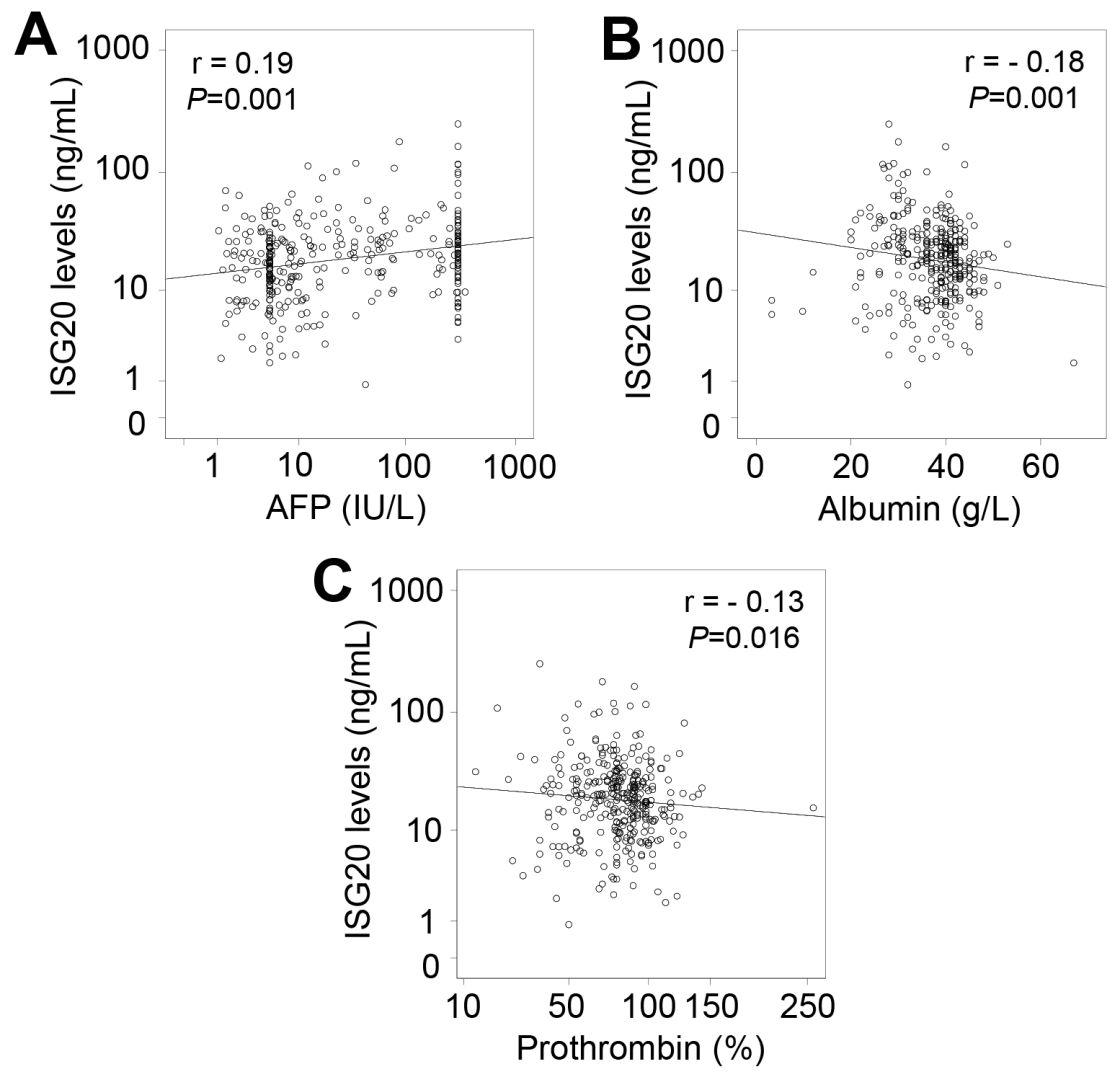
Correlation of ISG20 levels with clinical parameters in all patients with HBV-related liver diseases Correlations of ISG20 levels with different available clinical parameters were calculated by using Pearson correlation coefficient test. The Pearson’s r and *P* values are also presented. **(A)**: Comparison between ISG20 levels and alpha-fetoprotein (AFP) levels; **(B)**: Between ISG20 levels and albumin; **(C)**: Between ISG20 levels and prothrombin.

**Figure 5:**
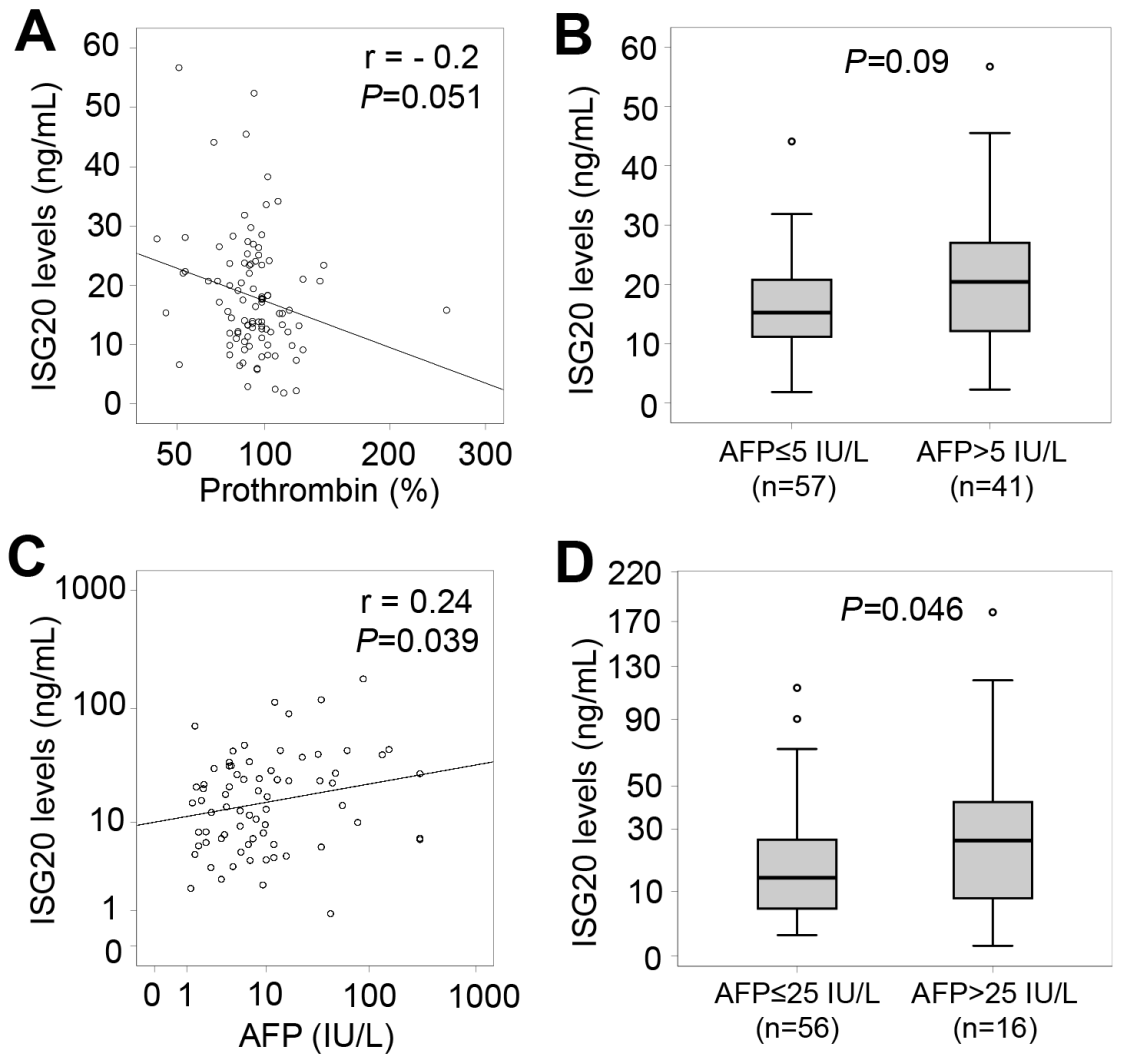
Relationship of ISG20 levels and clinical parameters in CHB and LC patients **(A)**: Correlation of ISG20 levels with prothrombin in CHB patients. **(B)**: CHB patients were classified based on alpha-fetoprotein (AFP) levels (either ≤5 IU/L or >5 IU/L), and ISG20 levels were compared between these classified subgroups. **(C)**: Correlation of ISG20 levels with AFP levels in LC patients. **(D)**: LC patients were classified based on AFP levels (either ≤25 IU/L or >25 IU/L), and ISG20 levels were compared between these classified subgroups. The correlations and corresponding *P* values were calculated by using Pearson correlation coefficient test. *P* values for comparisons were calculated by using linear regression adjusted for age and gender.

When analyzing correlations of ISG20 levels with clinical and laboratory parameters in all HBV patients, a weak correlation of ISG20 levels with AFP, albumin and prothrombin levels was observed (Figure 4). We also analyzed the correlations of ISG20 levels with clinical and laboratory parameters in each patient subgroup. In CHB patients, ISG20 levels were negatively correlated with prothrombin levels (Pearson’s r = -0.2, *P*=0.051) (Figure 5A). Although ISG20 levels were not correlated with AFP levels, ISG20 levels were slightly higher among CHB patients with AFP levels >5 IU/L compared to those with AFP levels ≤5 IU/L (20.4 ng/ml vs. 15.2 ng/ml, *P*=0.09) (Figure 5B). In LC patients, ISG20 levels were positively correlated with AFP levels (Pearson’s r = 0.24, *P*=0.039) and significantly higher in LC patients with AFP levels >25 IU/L compared to those with ≤25 IU/L (25.7 ng/ml vs. 13.6 ng/ml, *P*=0.046) (Figure 5C and 5D).

**Figure 6:**
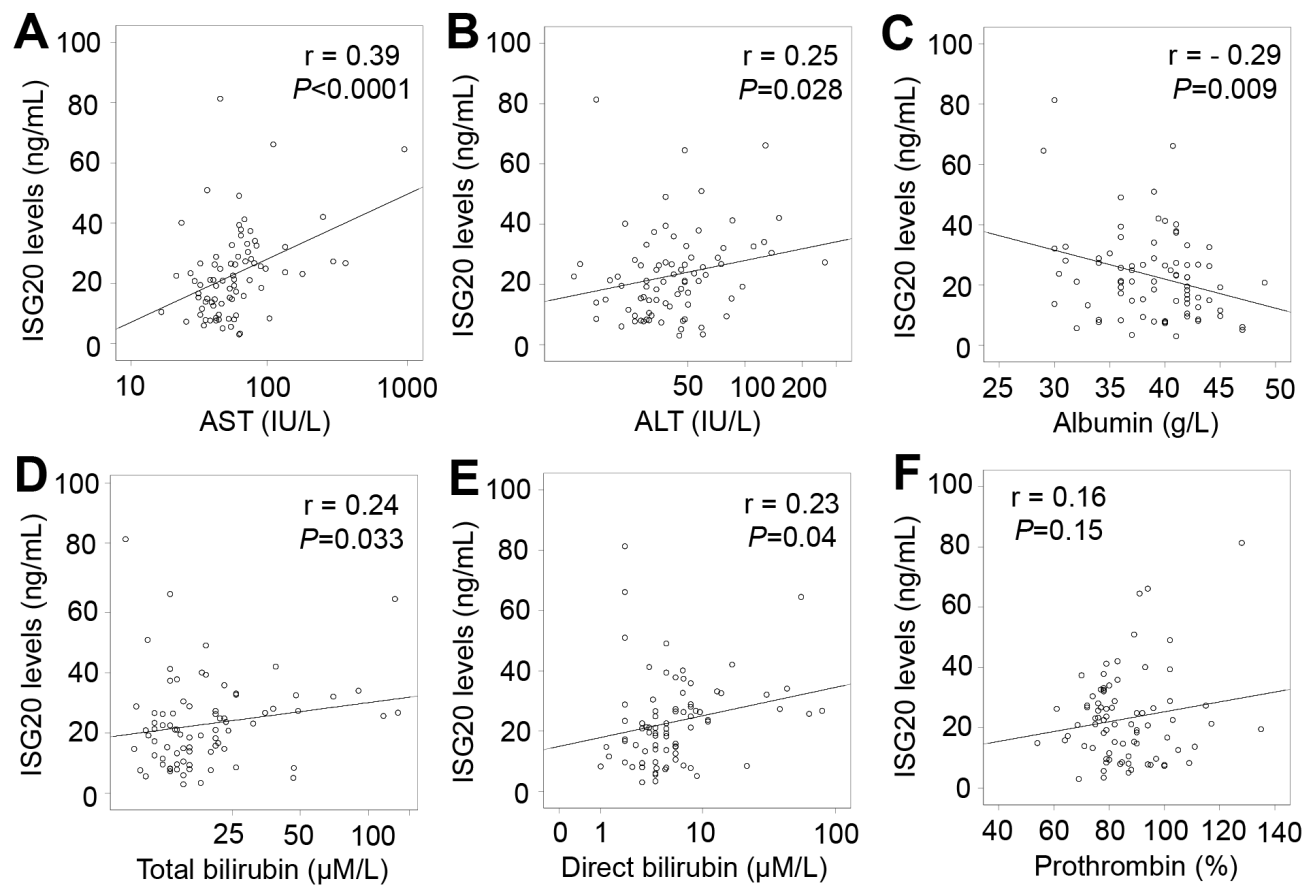
Correlation of ISG20 levels with clinical parameters in patients with only HCC Correlations of ISG20 levels with different available clinical parameters were calculated by using Pearson correlation coefficient test. The Pearson’s r and *P* values are also presented. **(A)**: Correlation between ISG20 levels and aspartate amino transferase (AST); **(B)**: Between ISG20 levels and alanine amino transferase (ALT); **(C)**: Between ISG20 levels and albumin; **(D)**: between ISG20 levels and total bilirubin; **(E)**: Between ISG20 levels and direct bilirubin; and **(F)**: Between ISG20 levels and prothrombin.

**Figure 7:**
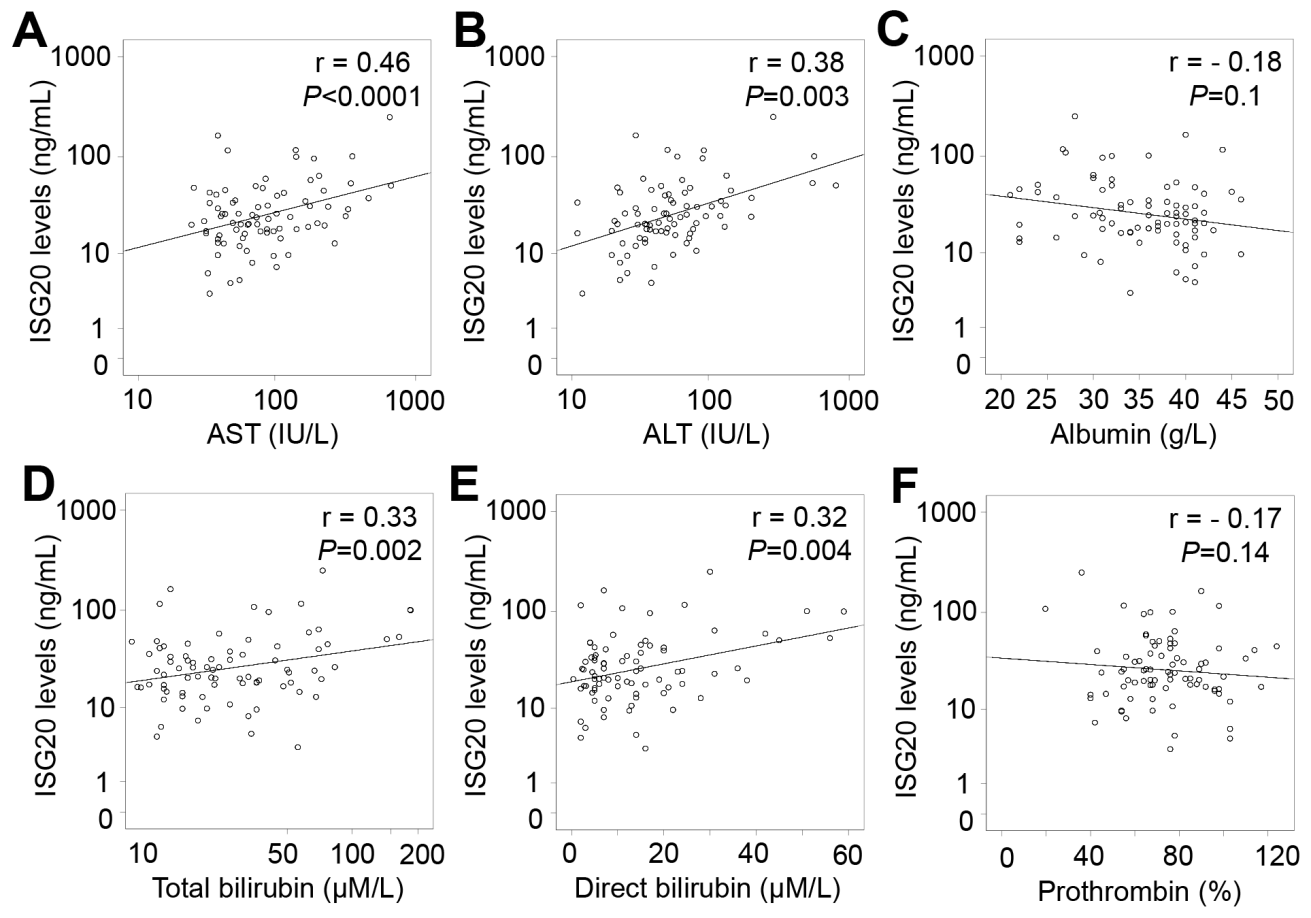
Correlation of ISG20 levels with clinical parameters in patients with LC plus HCC Correlations of ISG20 levels with different available clinical parameters were calculated by using Pearson correlation coefficient test. The Pearson’s r and *P* values are also presented. **(A)**: Correlation between ISG20 levels and aspartate amino transferase (AST); **(B)**: Between ISG20 levels and alanine amino transferase (ALT); **(C)**: Between ISG20 levels and albumin; **(D)**: Between ISG20 levels and total bilirubin; **(E)**: Between ISG20 levels and direct bilirubin; and **(F)**: Between ISG20 levels and prothrombin.

**Figure 8:**
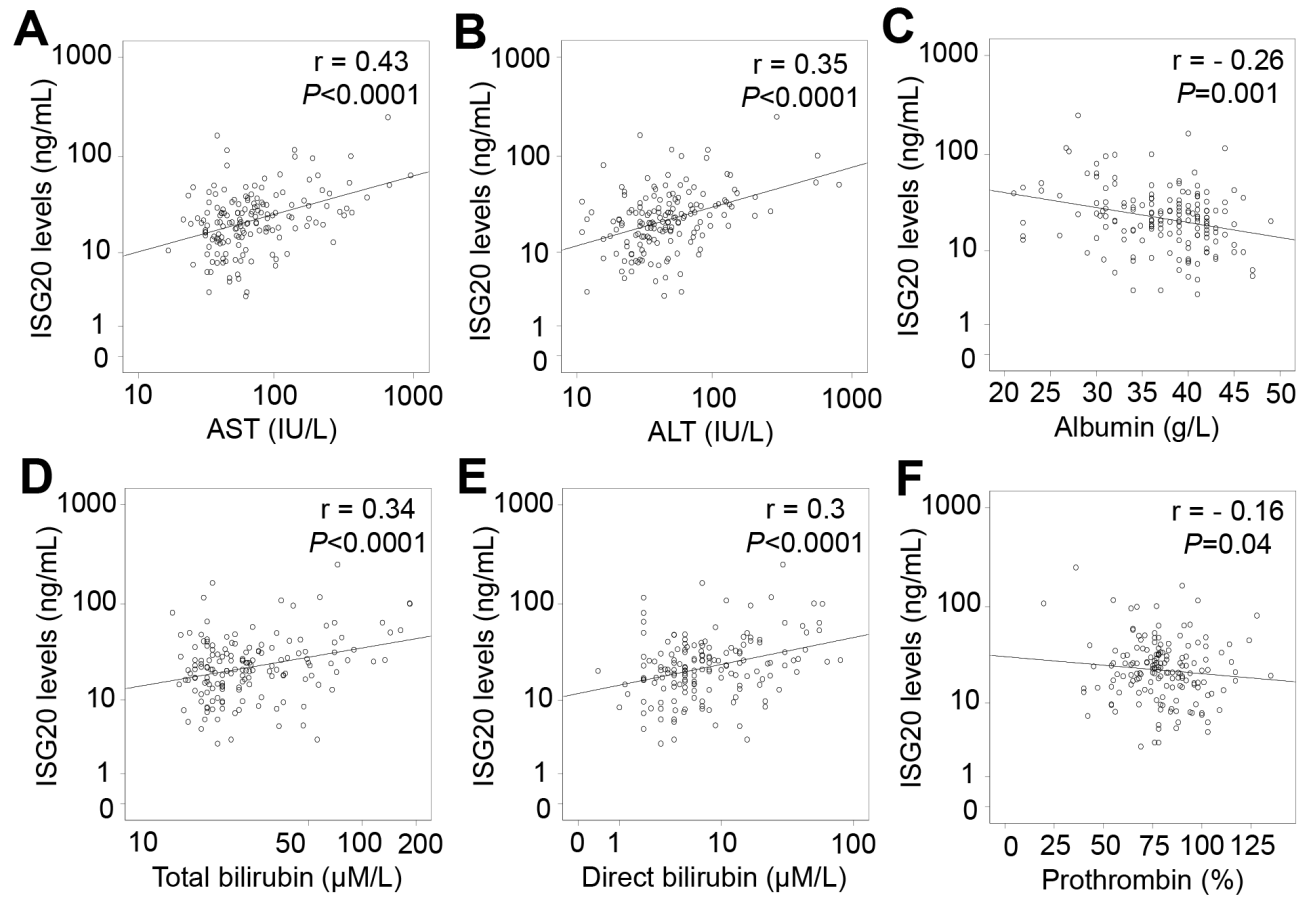
Correlation of ISG20 levels with clinical parameters in all HCC patients Correlations of ISG20 levels with different available clinical parameters were calculated by using Pearson correlation coefficient test. The Pearson’s r and *P* values are also presented. **(A)**: Correlation between ISG20 levels and aspartate amino transferase (AST); **(B)**: Between ISG20 levels and alanine amino transferase (ALT); **(C)**: Between ISG20 levels and albumin; **(D)**: Between ISG20 levels and total bilirubin; **(E)**: Between ISG20 levels and direct bilirubin; and **(F)**: Between ISG20 levels and prothrombin.

In patients with only HCC, ISG20 levels were positively correlated with liver function parameters including AST, ALT, total and direct bilirubin levels (Pearson’s r = 0.39, 0.25, 0.24, and 0.23; *P*<0.0001, =0.028, 0.033 and 0.04, respectively), and negatively correlated with albumin levels (Pearson’s r = -0.29; *P*=0.009) (Figure 6). In patients with LC plus HCC, ISG20 levels were strongly and positively correlated with AST, ALT, total and direct bilirubin levels (Pearson’s r = 0.46, 0.38, 0.33, and 0.32; *P*<0.0001, *P* = 0.003, 0.002 and 0.004, respectively) (Figure 7). In all HCC patients (patients with only HCC and those with LC plus HCC), we observed a strong correlation of ISG20 levels with AST, ALT, total and direct bilirubin levels (Pearson’s r = 0.43, 0.35, 0.34, 0.3; *P*<0.0001, respectively). ISG20 levels were also negatively correlated with albumin and prothrombin levels (Pearson’s r = -0.26, -0.16; *P*=0.001 and 0.04, respectively) (Figure 8).

## DISCUSSION

ISG20 is an exonuclease targeting RNA molecules and inhibiting replication of various RNA viruses, including HAV and HCV [16, 22]. Despite containing DNA in its genome, HBV replicates through reverse transcription of an RNA intermediate, the pregenomic RNA (pgRNA). Studies have shown that ISG20 inhibits HBV replication via degrading HBV-RNA molecules during infection [19, 20, 23]. Our previous study has also indicated the important role of *ISG15* genetic variants and ISG15 serum levels in HBV replication and the progression of HBV-related liver diseases [24].

To the best of our knowledge, this is the first study systematically investigating ISG20 levels in different conditions of HBV infection and its clinical outcome. We have shown that ISG20 levels are significantly modulated in patients with HBV-related liver diseases, especially in patients with HCC. ISG20 levels appear to be associated with the occurrence and development of HCC; the levels are significantly associated with liver function parameters in patients with HCC. The findings indicate that ISG20 plays an important role in the clinical outcome of HBV infection and may be involved essentially in the initiation and progression of HBV-related HCC.

ISG20 has been shown to be an important immune effector molecule in response to many infectious pathogens, in particular to hepatitis viruses [8, 16]. The present study has determined the serum levels of ISG20 in different forms of HBV-related liver diseases as well as in healthy individuals. We could demonstrate that ISG20 levels are strongly and negatively correlated with age in healthy individuals, but weakly positively correlated with age in HBV patients. No difference was observed between males and females both in patients and controls. The negative correlation of ISG20 levels with age in healthy individuals indicates aging of the immune system. The positive correlation observed in HBV patients may be due to the fact that the age of patients is higher in groups with advanced liver diseases and that the presence of advanced liver diseases certainly affects expression and production of ISG20.

ISG20 is expressed intracellularly and secreted by a wide range of cell types and organs such as peripheral blood leukocytes, vascular endothelial cells, lymph nodes, spleen, thymus, small intestine and lungs [9, 25, 26]. Higher ISG20 levels in HBV patients compared to healthy controls observed in this study are compatible with previous findings that ISG20 expression is induced not only by interferons, but also by double-stranded RNA [9, 11]. However, ISG20 expression is not efficiently induced by either the plasmid of the HBV full genome or the epsilon stem-loop part of HBV pgRNA in hepatocytes [19]. Higher *ISG20* expression at both the mRNA and protein levels were observed in the liver of HBV patients responding favourably to interferon-alpha treatment compared to non-responders and positively associated with response to interferon-alpha treatment [21, 27]. Therefore, up-regulation of ISG20 may be due to the array of interferons produced by immune cells in response to HBV infection, rather than due to HBV-derived DNA or RNA molecules. ISG20 has been shown to inhibit HBV replication by affecting HBV-RNA molecules [19, 20, 23]. However, we did not observe any correlation between ISG20 levels and HBV-DNA loads. This may be explained by the fact that intracellular ISG20 in hepatocytes has the capability to degrade HBV-RNA, but not extracellular ISG20 in serum; ISG20 in serum is mainly produced by distinct components of the immune system [9, 25]. As ISG20 is an exonuclease, the antiviral activity or immune regulatory functionality of serum ISG20 should be examined further.

While many immune genes, including *ISG20,* have been identified in hepatitis-induced HCC [28], the biological function of ISG20 in tumorigenesis remains poorly understood. Studies have documented an induction of *ISG20* in cervical and breast cancer cell lines [12] and up-regulation of *ISG20* mRNA in cervical tumours compared to normal cervix tissues [29]. Our study revealed that ISG20 levels are higher in HCC patients compared to those without HCC and increased according to the stages of HCC progression. In addition, *ISG20* mRNA expression is up-regulated in HCC tumour compared to adjacent non-tumour tissues. These results, which are compatible with a previous study showing overexpression of ISG20 mRNA in cancerous compared to adjacent tissues [13], indicate that ISG20 is involved in the development of HBV-related HCC. Differential expression patterns of ISG20 have been shown in the biopsies of various malignancies, including virus-related cancers like glioblastoma and HCC [30]. A recent study has shown that forced expression of ISG20 promotes metastases and angiogenesis via the IL-8/p-JAK2/p-STAT3 signalling pathway, both in liver cancer cell lines and in animal models, suggesting a pro-tumour role of ISG20 [13, 31]. However, the precise mechanism by which ISG20 triggers tumours and contributes to cancer development is far from being clear.

Importantly, our study shows that ISG20 levels are strongly associated with liver function tests in patients with HCC, also indicating that ISG20 may contribute to the pathogenesis of HBV-related HCC. In support of our observations, a previous study has shown that ISG20 overexpression in HCC tumours was correlated with clinical parameters, such as vascular invasion, AFP levels and tumour sizes, as well as with poorer recurrence-free survival in HCC patients [13]. In addition, ISG20 levels are correlated with AFP levels in LC patients and are different between CHB patients with AFP levels lower and higher than 5 IU/mL as well as between LC patients with AFP levels lower and higher than 25 IU/mL. These findings suggest that ISG20 levels are associated with the production of AFP in CHB and LC patients, which are high-risk groups for HCC development. Therefore, ISG20 levels may be considered a potential marker for HCC in addition to AFP levels in high-risk groups. However, further studies are required to examine the functional role and prognostic potential of ISG20 in HBV-related liver diseases, especially in tumorigenesis.

In conclusion, ISG20 levels are modulated during the progression of HBV infection and significantly associated with the clinical outcome of HBV-related liver diseases, especially in patients with HBV-related HCC. ISG20 levels may be considered as an additional indicator of liver function and clinical outcome of HBV-related HCC. Although our results show a potential role of ISG20 in HBV infection, the mechanisms by which ISG20 triggers HCC development and affects the clinical outcomes of HBV-related liver diseases need to be further elucidated.

## MATERIALS AND METHODS

### Patients and controls

Three hundred and thirty-nine Vietnamese HBV patients (n=339) enrolled at the 108 Military Central Hospital and the 103 Military Hospital, Hanoi, Vietnam were recruited for this study. The entire study cohort including healthy controls represent the Kinh ethnicity, the major ethnic group in Vietnam (86%). All patients were confirmed to be negative for HCV and HIV infections. HBV patients were diagnosed according to the European Association for the Study of the Liver (EASL) clinical practice guidelines for management of chronic HBV infection [32] and the American Association for the Study of Liver Diseases (AASLD) practice guideline for HCC [33]. The patients were categorized into three subgroups based on clinical manifestations, biochemical parameters, serological diagnosis, and histological examination. All patients had not been treated with interferons. The patient subgroups included chronic hepatitis B (CHB; n=100), patients with only liver cirrhosis (LC; n=79), and primary hepatocellular carcinoma patients (HCC; n=160). Patients with secondary HCC and HCC patients with metastases were excluded from this study. Among the 160 HCC patients, 81 patients presented with LC, and thus were assigned as a separate patient group with concomitant both LC and HCC (HCC+LC, n=81). Patients with LC were further categorized as Child-A, Child-B and Child-C subgroups based on the Child-Pugh scoring system [34]. Patients with HCC were further categorized as HCC stage-A, stage-B, stage-C and stage-D according to the Barcelona-Clinic Liver Cancer (BCLC) classification [35]. Laboratory parameters, namely aspartate transaminase (AST), alanine transaminase (ALT), total and direct bilirubin, prothrombin, albumin, alpha-fetoprotein (AFP) and HBV-DNA loads were measured by routine laboratory tests. In addition, we utilized 30 pairs of tumour and adjacent non-tumour tissue specimens collected from HCC patients who underwent surgery at the 108 Military Central Hospital to determine *ISG20* mRNA expression. HCC was confirmed by histology and the stage of tumour development was classified according to the BCLC classification [35]. The clinical profiles of these 30 HCC patients and data of the liver tissues have been described previously [36]. All specimens were frozen at -80°C until use.

Seventy-one healthy Vietnamese blood donors (HC; n=71) from the same ethnic group were included as control group. The control individuals were examined for their health status and confirmed to be negative for HBsAg, anti-HCV and anti-HIV antibodies. None of them had any chronic infectious disease or any other severe pathological condition nor a history of alcohol or drug use. Five ml of venous blood were collected from all participants and serum or plasma was separated from whole blood and used for the laboratory assays.

### Ethical statement

The study was approved by the Institutional Review Boards of the Vietnam Military Medical University (VMMU) and the 108 Military Central Hospital, Hanoi, Vietnam. All experiments were performed in accordance with relevant guidelines and regulations. Informed written consent was obtained from all participants or from their parents if subjects were less than 18 years after detailed explanation of the study at the time of blood sampling.

### Quantification of ISG20 serum levels by ELISA

ISG20 levels were measured in the serum samples from healthy controls and from patients with HBV-related liver diseases by using a commercially available interferon-stimulated gene 20 kDa protein (ISG20) ELISA Kit (MyBioSource, Inc., San Diego, CA, USA) following the manufacturer’s instructions. The principle of the assay is based on a competitive ELISA utilizing a polyclonal anti-ISG20 antibody and an ISG20 horseradish peroxidase (HRP) conjugate. Briefly, the microtiter plate provided in the ELISA kit had been pre-coated with a polyclonal anti-ISG20 antibody. 100 μL of standard protein ISG20, samples as well as phosphate-buffered saline (PBS) were put into the wells of the ELISA plate and 50 μL of ISG20-HRP conjugate were added, mixed properly and incubated for one hour at 37°C. The liquid of each well was removed and plates were washed five times with washing buffer. Subsequently, 50 μL of hydrogen peroxidase and TMB (3,3’,5,5’-Tetramethylbenzidine) substrate were added and the plate was incubated for 15 minutes at 37°C before stop solution was applied to terminate the reaction. Plates were immediately read at a wavelength of 450 nm by an ELISA reader. In order to calculate the concentrations of ISG20 protein, a standard curve was plotted based on the mean of O.D. (optical density) values and the known concentration of the standards. The concentrations of ISG20 were calculated based on the standard curve. The minimum detectable limit of ISG20 proteins was 0.1 ng/mL.

### Quantification of *ISG20* mRNA by RT-PCR

Total RNA was extracted from the 30 pairs of liver tissues with Trizol reagent (Life Technologies, Carlsbad, CA, USA), followed by reverse transcription of RNA into cDNA (QuantiTect Reverse Transcription Kit; Qiagen, Hilden, Germany). *ISG20* mRNA levels were determined through quantification of *ISG20* cDNA by qRT-PCR using SYBR Green PCR master mix (Bioline, Luckenwalde, Germany). The *GAPDH* gene (glyceraldehyde-3-phosphate dehydrogenase) was used as a reference. Primer sequences applied were ISG20_F: 5’- ATC TCT GAG GGT CCC CAA GGA -3’ and ISG20_F: 5’- TTC AGT CTG ACA CAG CCA GGC G -3’, GAPDH_F: 5’- CCA CCC ATG GCA AAT TCC ATG GCA -3’ and GAPDH_R: 5’- TCT AGA CGG CAG GTC AGG TCC ACC -3’ [26]. Thermal cycling conditions were 2 min 95°C, followed by 45 cycles of 95°C, 5 sec and annealing and extension at 58°C for 20 sec. Reaction specificity was confirmed by melting curve analyses starting from 58°C to 85°C. All qRT-PCR reactions were performed in duplicate and were repeated twice (LightCycler^®^ 480 real-time PCR system; Roche, Basel, Switzerland). The fold change of *ISG20* mRNA was normalized based upon the ΔCt method against the expression of *GAPDH*.

### Statistical analysis

Clinical and demographic data are given in medians with ranges for quantitative variables and categorical data given as numbers with percentages. Categorical variables were compared using Chi-square or Fisher’s exact tests. Student’s *t*-test and Mann Whitney Wilcoxon test were used to compare parametric and non-parametric data of quantitative variables between two groups, respectively. Kruskal-Wallis test was used to compare non-parametric data of quantitative variables among more than two groups. Paired-samples *t*-test was used to compare *ISG20* mRNA levels expressed in tumour and adjacent non-tumour tissues. A linear regression model was applied to analyze the relationship of clinical parameters of HBV patients and ISG20 levels adjusted for the confounding effects of age and gender. Pearson´s correlation coefficient test was used to analyze correlations between ISG20 levels and clinical parameters of HBV patients. All statistical analyses were performed using the SPSS software (SPSS Statistics, IBM, Armonk, NY, USA). The level of significance was set at a *P* value <0.05.

### Key points

- ISG20 is an exonuclease targeting RNA molecules and inhibiting hepatitis viruses.

- Varying ISG20 levels were associated with different forms of HBV-related liver diseases.

- ISG20 levels were higher in patients wiFth HCC compared to those without HCC.

- ISG20 levels were correlated with liver function tests among HCC patients.

- ISG20 might be a potential indicator clinical outcome in HBV-related HCC.

## Abbreviations

CHB: chronic hepatitis B
HBV: hepatitis B virus
HC: healthy control
LC: liver cirrhosis
HCC: hepatocellular carcinoma
ISG20: Interferon-stimulated gene 20 kDa protein
